# Identifying and overcoming barriers to participation of minority populations in clinical trials: Lessons learned from the VanDAAM study

**DOI:** 10.1002/cam4.5000

**Published:** 2022-07-07

**Authors:** Angelina K. C. Fink, Amanda C. DeRenzis, Shivanshu Awasthi, Nawreen Jahan, Peter A. S. Johnstone, Julio Pow‐Sang, Javier Torres‐Roca, Daniel Grass, Daniel Fernandez, Arash Naghavi, Susan Tan, Brandon Manley, Roger Li, Michael Poch, Alice Yu, Nikki Little, Eppie Bass, Cesar E. Ercole, Evangelia Katsoulakis, Ryan Burri, Riley Smith, Nathanael B. Stanley, Susan T. Vadaparampil, Kosj Yamoah

**Affiliations:** ^1^ Cancer Epidemiology H. Lee Moffitt Cancer Center & Research Institute Tampa Florida USA; ^2^ Tallahassee Memorial Hospital Tallahassee Florida USA; ^3^ Radiation Oncology H. Lee Moffitt Cancer Center & Research Institute Tampa Florida USA; ^4^ Genitourinary Oncology H. Lee Moffitt Cancer Center & Research Institute Tampa Florida USA; ^5^ Urology Section James A. Haley Veterans' Hospital Tampa Florida USA; ^6^ Radiation James A. Haley Veterans' Hospital Tampa Florida USA; ^7^ Radiation Bay Pines VA Healthcare System Bay Pines Florida USA; ^8^ Office of Community Outreach, Engagement & Equity (COEE) H. Lee Moffitt Cancer Center & Research Institute Tampa Florida USA; ^9^ Health Outcomes & Behavior H. Lee Moffitt Cancer Center & Research Institute Tampa Florida USA

**Keywords:** African American men (AAM), Decipher, minority populations, prostate cancer (PCa), VanDAAM

## Abstract

Participation in cancer research trials by minority populations is imperative in reducing disparities in clinical outcomes. Even with increased awareness of the importance of minority patient inclusion in clinical research to improve cancer care and survival, significant barriers persist in accruing and retaining minority patients into clinical trials. This study sought to identify and address barriers to minority accrual to a minimal risk clinical research study in real‐time.

## Graphical abstract

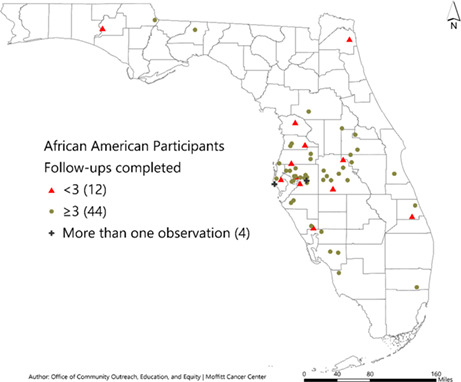

## 1 INTRODUCTION

Participation in clinical trials is integral to the advancement of treatments and improvement of outcomes and quality of life for patients with cancer. Prostate cancer (PCa) accounts for more than 1 in 5 new cancer diagnoses in men and continues to be one of the leading causes of cancer‐related death.^1^ African‐American men (AAM) have higher PCa incidence and mortality rates than non–African‐American men (NAAM), with a 2.5‐fold higher risk of being diagnosed with a more aggressive form of PCa.^2^ AAM continues to endure a disproportionate cancer burden, having the highest death rate and shortest survival of any racial or ethnic group for most cancers.^3^ AAM are more likely to be diagnosed at a younger age, have more aggressive disease, and have higher mortality rates than NAAM.^4^ Additionally, the clinical course of disease, serum prostate‐specific antigen (PSA) levels, and social factors differ significantly between AAM and their NAAM counterparts.^5^ The combination of these factors with social determinants of health and disparate access to care predispose AAM to face greater health challenges and increased burden throughout the continuum of PCa clinical care.

Facilitating minority population participation in clinical trials is one approach to mitigating these disparities. Ensuring that populations who experience the greatest cancer burden are represented in trials will advance treatment options to achieve the best possible long‐term clinical outcomes. Despite the importance of inclusion of minority patients in clinical trials, barriers continue to result in underrepresentation of these populations. Reasons for this disparity gap among AAM have been previously identified in the literature and include, but are not limited to, a continuing lack of trust in the medical community, costs of participation, and fear of unethical conduct of research stemming from historical mistreatment and abuse, such as the Tuskegee syphilis trial.^6^ Additionally, concerns regarding confidentiality, additional burden, privacy, and stigmas associated with research participation within the African American community further accentuate the disparity gaps in recruitment to clinical trials.^7^ Finally, prior research has shown that implicit bias from clinicians may preclude patients from receiving healthcare or access to clinical trials based on characteristics such as race or gender.^8^ These factors result in underenrollment and lack of representation of minority populations in clinical trials, which contributes to observed racial disparities in clinical outcomes. The goal of this study was to identify and mitigate barriers to the enrollment and retention of AAM patients on a minimal‐risk clinical study.

## 2 METHODS

Barriers to the enrollment and retention of AAM identified during the accrual and follow‐up on a minimal risk, prospective validation clinical study titled “A Validation Study on the Impact of Decipher® Testing on Treatment Recommendations in African American and Non‐African Men with Prostate Cancer (VanDAAM) – NCT#0272373” will be discussed for consideration in the development of future clinical trials. The study coordinator documented reasons provided by patients for declining study participation on the VanDAAM trial at Moffitt Cancer Center (MCC).

The VanDAAM study was a multisite validation study of the genomic classifier Decipher® in AAM. The Decipher® score, a CLIA‐approved genomic test that predicts aggressive disease and risk of distant metastases in men with localized PCa, was developed mostly with patients of European descent. Prior to VanDAAM, Decipher® had not been formally prospectively validated for use among AAM. Patients were recruited to the VanDAAM study on a 1:1 (AAM:EAM) basis and matched based the Cancer of the Prostate Risk Assessment (CAPRA) score,^9^ which includes age at diagnosis, Gleason score, staging, and percentage of positive biopsy cores from a baseline prostate biopsy. Patients with low‐ or intermediate‐risk PCa were screened for enrollment. Patients who elected active surveillance were ineligible for participation. Decipher® testing was ordered for all patients on their biopsy and/or radical prostatectomy tumor tissue. Participants were followed for 2 years after treatment to collect data on their clinical outcomes to determine the accuracy of the Decipher® score in predicting aggressive disease, defined as PSA failure within 2 years in AAM.

## 3 SETTING AND DESIGN

Primary recruitment for the VanDAAM study took place at three large clinical care settings in West Central Florida. MCC is a large NCI‐Designated Comprehensive Cancer Center serving a 15‐county catchment area. The James A. Haley Veterans' Hospital and Bay Pines VA Healthcare System served as secondary recruitment sites. All recruitment sites are located in the Tampa Bay area in Florida in Hillsborough and Pinellas counties, which have the largest racially and ethnically diverse population in West Central Florida. The study protocol involved completing a baseline assessment and six follow‐up visits at 3, 6, 9, 12, 18, and 24 months (±2 months) after treatment; these visits were strategically planned to occur in line with routine clinical care visits to minimize patient burden and increase study visit compliance.

### 3.1 Screening to increase accrual

As part of a dynamic real‐time monitoring process, the research team determined that tailoring their approach to identifying and consenting patients for the study was necessary to successfully meet the prespecified enrollment requirement for minority patients within the study timeline. As such, provider schedules for both the genitourinary (GU) surgical and GU radiation oncology clinics were carefully prescreened, and AAM patients who met the general eligibility requirements based on their available medical records were flagged to be approached at the time of their visit.

The goal of this approach was to offer study participation to every newly diagnosed treatment‐naïve low‐, intermediate‐risk AAM patient with PCa. Clinical providers were notified in advance of potential study patients and research staff would be in the clinic and available to provide more information to interested patients. Additionally, flyers with information regarding the study and contact information were posted in clinicians' workspaces to encourage clinical staff to refer patients for study participation.

## 4 RESULTS

### 4.1 Accrual

**FIGURE 1:**
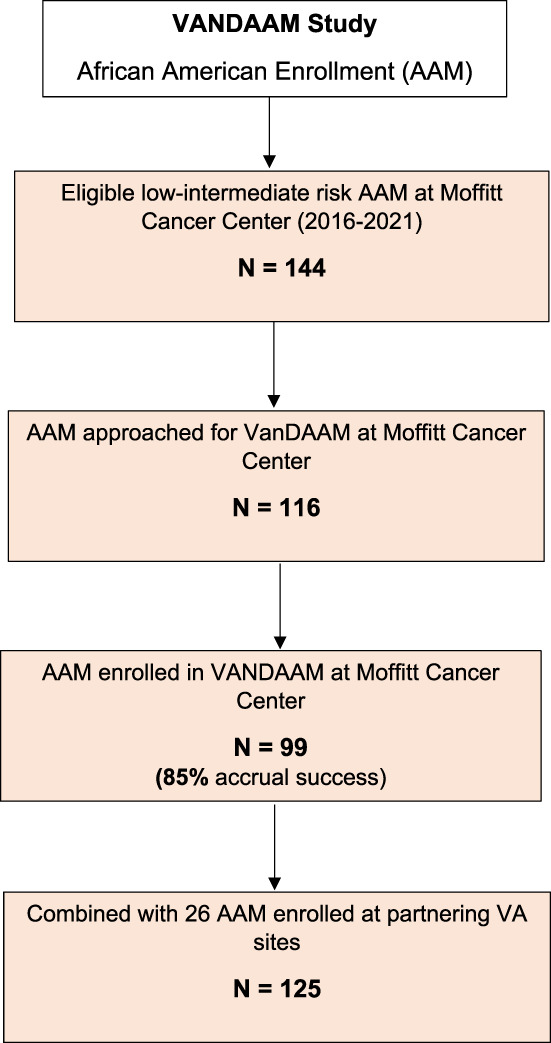
Accrual consort diagram. *Total number and reason for declinations was documented for patients enrolled at Moffitt Cancer Center only.

Recruitment for the VanDAAM study occurred between April 2016 and May 2021, with an accrual goal of 250 patients: 125 AAM and NAAM matched pairs. Of the 144 AAM patients with primary PCa who were seen between 2016 and 2021 by the GU oncology programs at MCC, the study team approached 116 AAM and enrolled 99 AAM, resulting in an accrual success rate of 85% among those approached for participation. An additional 26 AAM were enrolled at partnering VA sites to meet the accrual goal of 125 AAM patients (Figure 1). Data on the number of patients who were approached and declined participation and their reasons for declination were not captured at the two enrolling VA sites.

Among all of the AAM patients approached for study participation at MCC (*N* = 116), reasons for declining participation included being overwhelmed or hesitant to participate in research (*N* = 5), being uninterested in participating in research (*N* = 4), having an undecided treatment plan or electing for active surveillance (*N* = 3), the reason for declination was not provided (*N* = 3), and insurance concerns (*N* = 2). The main reason that NAAM patients declined participation was concern associated with the billing of insurance (*N* = 5). Only 1 NAAM patient stated that they were hesitant to participate in research versus 5 AAM patients (Table 1). Of AAM patients who declined study participation, 56% (*N* = 10) had at least some college or trade school education versus 59% (*N* = 74) of consented AAM. For NAAM who declined participation, 40% (*N* = 6) had at least some college or trade school versus 74% (*N* = 87) of NAAM patients who consented to participate.

**TABLE 1:** Reasons for declining study participation

Reported reason	AAM (*N* = 17/125)	NAAM (*N* = 15/118)
Overwhelmed or hesitant to participate	5 (4%)	1 (1%)
Not interested in research participation	4 (3%)	3 (2%)
Treatment undecided	3 (2.5%)	2 (1.5%)
Reason not provided	3 (2.5%)	3 (2%)
Billing of insurance	2 (2%)	5 (4%)
Personal reasons	0 (0%)	1 (1%)

Abbreviations: AAM, African American Men; NAAM, non‐African American Men.

AAM who declined because of hesitation to participate or being uninterested in research participation identified two main reasons for their hesitancy or disinterest. First, hesitation was due to the patient's knowledge of historically unethical research conduct. Some AAM specifically mentioned these types of events as the reason they were hesitant to participate at the time they were approached for the VanDAAM study. Secondly, the influence of family members or caregiver's concerns about the study played a major role in patients' decision‐making process. In several consenting encounters, patients initially showed interest and willingness to participate in the study, but when their family members demonstrated concern or hesitation, the patient would question their decision to participate and their knowledge and/or understanding of the purpose of the study, resulting in a loss of trust with the research team, leading to refusal to participate.

### 4.2 Retention

Approximately 67% of the AAM patients (*N* = 66/99) and 51% of the NAAM patients (*N* = 61/118) recruited at MCC have completed the 2‐year posttreatment follow‐up period at the time of this report. Twenty‐two AAM and 48 NAAM patients are still on active follow‐up, per the study protocol. Eleven AAM and nine NAAM were removed from the study prior to the follow‐up period because Decipher® testing was not performed, the patient had no available treatment data, the patient withdrew from the study, the patient experienced screen failure, or the patient died.

**FIGURE 2:**
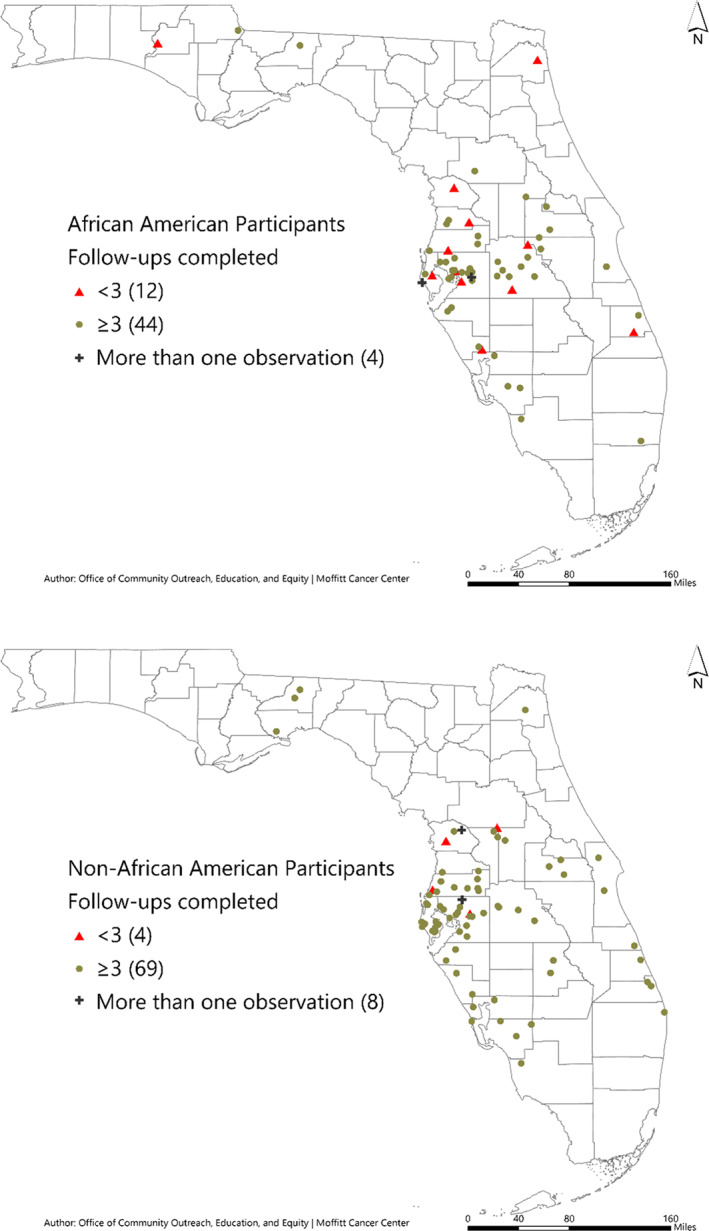
Follow‐up completion by zipcode.

The VanDAAM study was designed such that the study visits closely matched with NCCN follow‐up guidelines and real‐world practice patterns. Of patients who have completed the follow‐up period, 91% (*N* = 60) of AAM and 93% (*N* = 57) of NAAM completed PSA tests, per NCCN guidelines. Only 26% AAM (*N* = 17) and NAAM (*N* = 16) have completed all planned study follow‐ups with documented PSA levels. Forty‐one percent (*N* = 7) of AAM patients who completed all follow‐up visits were living within Hillsborough County, where MCC is located, vs 38% of NAAM. Of AAM patients who completed ≤3 PSA follow‐ups (*N* = 14), 64% live outside of Hillsborough County (*N* = 9) vs only 25% of their NAAM counterparts (*N* = 15). Despite the implementation of additional intervention methods focused on patient retention, AAM (*N* = 14 [17%]) were still more likely than their NAAM (*N* = 6 [6%]) counterparts to complete less than 3 PSA tests 2 years posttreatment in follow‐up on the VanDAAM study (Figure 2).

The majority of both AAM (*N* = 70 [83%]) and NAAM (*N* = 85 [81%]) enrolled in the study resided within MCC's15‐county catchment area spanning West Central Florida. Patients who completed ≤3 follow‐ups [AAM, *N* = 11 (79%) and NAAM, *N* = 5 (83%)], mainly resided within MCC's15‐county catchment area, and compliance did not differ by race. However, living over 50 miles from MCC did pose a challenge for the retention of AAM (*N* = 8 [8%]), whereas it had no impact on the retention of NAAM.

## 5 DISCUSSION

### 5.1 Accrual

Similar to prior studies, hesitancy to participate in clinical research was the most common reason for AAM patients declining participation in the VanDAAM study. One of the most commonly reported barriers encountered in the accrual of AAM patients to clinical trials is the lack of trust between the patient and researchers.^10^ Personal and family beliefs based on historical events influence AAM patients' willingness to enroll and participate in clinical trials, including minimal‐risk non‐therapeutic studies, such as the VanDAAM study. It has been suggested that AAM who are aware of trials such as the Tuskegee study are less likely to trust medical researchers and participate in trials.

Conversely, other research has suggested that historical unethical research, such as the Tuskegee trial, had no effect on the recruitment of AAM patients into research trials.^11^ Our experience with the VanDAAM study provides further evidence to support prior research indicating that historical events continue to influence minority populations' hesitancy to participate in research. Even though patients were informed that participating in the VanDAAM study would not impact their clinical care, these beliefs stemming from historical events still caused some AAM patients to be unwilling to participate.

### 5.2 Role of social support in participation

Social support and physician recommendations have also been identified as contributing factors in the recruitment of AAM patients.^12^ During the informed consent process for the VanDAAM study, many patients were accompanied by family or friends. Similar to prior research, AAM who were accompanied by family or friends were more likely to be hesitant or decline to participate in the VANDAAM study if their companion did not show interest or concur with study participation. In these situations, additional time and education on study participation were needed for either the patients or their companion to make them more comfortable with the consenting process. These data suggest that additional resources and time dedicated to clinical trial education for both patients and family members is critical to improve clinical trial participation among minority patients.

### 5.3 Impact of clinician's knowledge and study support

Though the role of physicians' implicit bias has been extensively documented as a factor contributing to the lower accrual of minority populations to clinical studies, the VanDAAM study did not specifically find this to be a factor that impacted the accrual of AAM. Conversely, it was found that, once the clinical teams in the GU and Radiation Oncology clinics at MCC were made aware of the study's purpose and eligibility criteria, they became an integral part of the study enrollment process. Once the clinical teams understood the study requirements, they often reached out to the study team to proactively recommend potential patients for consideration.

To further increase patients' comfort with and knowledge regarding the trial, the study team engaged treating physicians and/or advanced practicing professionals (APPs) to introduce and provide key details regarding the study before the research coordinator approached the patient. Prior research has shown that clinical providers' support of clinical trials and their ability to accurately describe the study in culturally sensitive terms to patients with different educational and health literacy levels greatly improved clinical trial enrollment^13^; this also proved to be the case for the VanDAAM study. Educating physicians and APPs on the study and having them demonstrate support for the research and provide additional education directly to AAM patients increased the likelihood of AAM patients consenting to the VanDAAM study. With integration of physicians and APPs into the education and consenting process, a clear difference was seen in AAM study participation when compared with instances in which the physician or APP was not familiar with the study and was unable to have a detailed discussion with the patient regarding participation.

Our study team also reported that many AAM cited being overwhelmed as a major reason for declining study participation. It was determined that patients were more hesitant to participate and more likely to defer participation when coordinators approached them before they saw a physician for their clinical appointment. Patients were often more comfortable deciding to participate in research once they had a treatment plan confirmed and scheduled with their providers. As a result, the recruitment process was modified to only approach patients after they had been seen by their clinical provider. Coupled with integrating the clinical team into the education of the study approach, this procedure yielded an 85% success rate in accruing AAM patients to the VanDAAM study.

### 5.4 Insurance concerns

Per the VanDAAM protocol and contractual agreements with Decipher Biosciences, patients did not incur any out‐of‐pocket costs for their participation on the VanDAAM study. Despite this agreement, NAAM were still more likely to be hesitant or declined participation due to the billing of insurance. Some patients had the misconception that their insurance company would know that they were participating in a research study and would receive information regarding the patient's Decipher® results. There was particular concern that their genetic information and results would be used against them for future healthcare needs. Although general education on the purpose of Decipher® testing was needed for most participants, AAM patients tended to be more comfortable and verbalize understanding of the study purpose after additional explanation was provided by research and/or clinical teams.

Previous studies have suggested that AAM patients are half as likely to participate in research if they only had a high school diploma or less education.^14^ In line with these findings, most AAM who consented to the VanDAAM study had some amount of higher education than the AAM who declined. Higher education may have assisted patients to fully understand the difference between genetic testing to determine inheritable risk of cancer vs personalized tumor genomic testing, such as Decipher®.

### 5.5 Retention

Current NCCN guidelines recommend patients visit a clinical provider for a PSA blood test every 6–12 months for the first 5 years after treatment and annually thereafter.^15^ The VanDAAM protocol was designed so that study visits closely matched with standard‐of‐care follow‐up guidelines and real‐world practice patterns. The protocol‐defined follow‐up schedule was provided as a recommendation to physicians but was not mandated. Throughout the duration of the study, clinical guidelines and practice for low‐ to intermediate‐risk PCa changed slightly, potentially affecting adherence to protocol follow‐up schedules. Less than 30% of both AAM and NAAM met the recommended protocol schedule of six PSA evaluations during the 2‐year follow‐up period.

AAM patients who participated in the study were more likely to not complete the clinically recommended follow‐up schedule after their treatment for PCa than their NAAM counterparts without intervention from the research team. This is consistent with previous studies that analyzed SEER data cohorts and found the overall median follow‐up time to be for PCa of 5.3 years for non‐Hispanic, White patients but only 4.8 years for AAM.^16^ Although the protocol‐defined follow‐up schedule for PSA evaluations had a lower retention rate, 91% (*N* = 60) of AAM and 93% (*N* = 57) of NAAM who completed their follow‐up for the VanDAAM study met the current NCCN guidelines for PSA follow‐up after treatment.

As part of participation on the VanDAAM study, patients were asked to complete quality of life questionnaires, which were often completed clinically as part of standard of care (American Urological Association [AUA] and Expanded Prostate Cancer Index [EPIC] questionnaires). A significant decrease in the completion of follow‐up visits was noted among AAM 3–6 months after treatment without intervention from the research team. Follow‐up completion rates 6–9 months after treatment were approximately 75% for AAM versus approximately 85% for NAAM. To ensure participants successfully completed follow‐up visits and meet the study endpoint, additional intervention was required from the research team, including reaching out to the patients directly to complete questionnaires remotely and initiating contact with their clinical providers to provide assistance in scheduling their PSA evaluations and/or obtaining the results (with the patient's permission). Of the AAM contacted, 36 questionnaires were completed by phone that otherwise would have been missed. Even with additional attempts by the research team, 8 AAM patients were lost to follow‐up vs only 2 NAAM.

Most AAM and NAAM patients resided within the state of Florida. Given the similar geographical locations of both populations, distance may not be suggestive as the primary reason for not receiving clinical care, as per NCCN guidelines. Even with additional interventions and similar geographical locations, AAM were still less likely to meet NCCN guidelines during their follow‐up visits while enrolled on VanDAAM than their NAAM counterparts. However, for patients who resided over 50 miles from the treating institution, distance did seem to pose a greater challenge for AAM than NAAM in the completion of both clinical care and study follow‐up visits.

### 5.6 Strengths and limitations

Strengths of this study included the development and implementation of multiple strategies in real time to increase enrollment and retention of a minority population to a minimal‐risk clinical research study. This was a targeted enrollment study in which the study team extensively prescreened all GU and Radiation Oncology clinic schedules at MCC to ensure that all AAM patients with clinical visits were identified and approached for study participation. Additionally, all study team members received supplementary training on cultural competency and hands‐on supervised sessions on how to discuss clinical studies and appropriately provide informed consent to participants from minority populations. This targeted accrual, coupled with additional training for the study team, including the providers, was central to the accrual success of this study. These strategies are now being implemented to increase minority accrual across various types of clinical trials. Additionally, cohesive teams were built through the conduct of this study, which has resulted in increased awareness and knowledge of potential clinical trials that may be available to minority participants. Also, a minority accrual target goal is now being more frequently discussed as an integral part of future clinical trial study design at MCC.

Conversely, limitations of this study included the fact that there was no structured data collection form used to capture the reasons for lack of study participation. However, reasons were documented as free text and were logged in the screening database accordingly so that patients who declined participation were not approached at future appointments. Future trials will use a structured data collection instrument detailing reasons for declination to further refine recruitment strategies to increase minority accrual.

Another limitation was that the tailored approach to screening and approaching patients, the thorough education of clinical and research teams, and the intensive follow‐up may not always be financially feasible. Furthermore, the shortage and decreasing number of available clinical trial personnel per study may make this approach infeasible in some settings. Even with a tailored real‐time approach to screening, 28 AAM patients were not approached for participation and eight AAM patients were lost to follow‐up. Additional discussion and process adjustments may be needed to ensure that all eligible minority patients are approached for clinical trial participation.

## 6 CONCLUSION

Participation in cancer research trials by minority populations is imperative in reducing disparities in clinical outcomes. Even with increased awareness of the importance of minority patient inclusion in clinical research to improve cancer care and survival, significant barriers persist in accruing and retaining minority patients into clinical trials. Although the VanDAAM study was successful in enrolling AAM patients with PCa, it highlighted known barriers to the accrual of minorities into clinical trials and provided prospective validation for several proposed implementation strategies for increasing minority accrual to clinical research.

Mistrust of the medical and research community continues to pose significant challenges to engaging minority populations in clinical research. As identified through the VanDAAM study, additional support and education from both healthcare providers and research personnel were necessary to ensure AAM patients understood the purpose and details of the study and were comfortable with their decision to participate. One of the strengths of the VanDAAM study was the engagement of a very diverse team of research and clinical providers who represented many different ethnic and racial groups and were supporting and actively participating in the enrollment of AAM to this study. Additionally, research coordinators consenting to this study received extensive training on cultural competence and awareness in respect to recruiting minority populations.

To close the disparity gap of representation of minority populations in clinical trials, strategies should begin in the earliest stages of study development, starting with the inclusion of a specific minority accrual target. The VanDAAM study was successful in recruiting 125 AAM due, in part, to this being a required goal of the study. Additionally, engagement and support from the clinical teams by providing them with detailed knowledge and a role in educating their patients on the study is imperative for gaining the trust of patients who may otherwise be hesitant and unlikely to participate. Closely monitoring and engaging patients during the follow‐up period and planning for additional research staff time and resources to retain patients and meet study endpoints may enhance successful study completion. Addressing barriers to accrual and retention of minority populations on multiple levels early in the course of the study timeline may lead to increased representation of minorities in clinical research, thereby providing more personalized treatment options and improving future clinical outcomes.

## AUTHOR CONTRIBUTIONS

Kosj Yamoah; Angelina K.C. Fink and Shivanshu Awasthi: Study design. Kosj Yamoah: Study Supervision. Shivanshu Awasthi, Angelina K.C. Fink, Nathanael B. Stanley: Data Analysis. Kosj Yamoah, Angelina K.C. Fink, Amanda C. DeRenzis, Shivanshu Awasthi: Writing Original draft. All the authors involved in the critical revision and approval for submission.

## CONFLICT OF INTEREST

The authors have no conflicts of interest to report.

## ETHICS STATEMENT

Full Institutional Review Board approval was received prior to study conduct. Moffitt Cancer Center; Advarra (#Pro00016323), James A. Haley Veterans' Hospital; University of South Florida IRB (#Pro00031822), and Bay Pines Veterans' Hospital; Bay Pines VAHCS IRB (#02976).

## Data Availability

The datasets generated during and/or analyzed during the current study are available from the corresponding author on reasonable request.
